# Simultaneous Determination and Pharmacokinetic Study of Quercetin, Luteolin, and Apigenin in Rat Plasma after Oral Administration of* Matricaria chamomilla* L. Extract by HPLC-UV

**DOI:** 10.1155/2017/8370584

**Published:** 2017-03-08

**Authors:** Xiaoxv Dong, Wei Lan, Xingbin Yin, Chunjing Yang, Wenping Wang, Jian Ni

**Affiliations:** ^1^Beijing University of Chinese Medicine, Beijing 100029, China; ^2^Xinjiang Key Laboratory of Famous Prescription and Science of Formulas, Urumqi 830011, China

## Abstract

A simple and sensitive HPLC-UV method has been developed for the simultaneous determination of quercetin, luteolin, and apigenin in rat plasma after oral administration of* Matricaria chamomilla *L. extract. The flow rate was set at 1.0 ml/min and the detection wavelength was kept at 350 nm. The calibration curves were linear in the range of 0.11–11.36 *μ*g/ml for quercetin, 0.11–11.20 *μ*g/ml for luteolin, and 0.11–10.60 *μ*g/ml for apigenin, respectively. The intraday and interday precisions (RSD) were less than 8.32 and 8.81%, respectively. The lower limits of quantification (LLOQ) of the three compounds were 0.11 *μ*g/ml. The mean recoveries for quercetin, luteolin, and apigenin were 99.11, 95.62, and 95.21%, respectively. Stability studies demonstrated that the three compounds were stable in the preparation and analytical process. The maximum plasma concentration (*C*_max_) was 0.29 ± 0.06, 3.04 ± 0.60, and 0.42 ± 0.10 *μ*g/ml, respectively. The time to reach the maximum plasma concentration (*T*_max_) was 0.79 ± 0.25, 0.42 ± 0.09, and 0.51 ± 0.13 h, respectively. The validated method was successfully applied to investigate the pharmacokinetics study of quercetin, luteolin, and apigenin in rat plasma after oral administration of* M. chamomilla *extract.

## 1. Introduction

*Matricaria chamomilla* L. (German chamomile), a member of the Asteraceae family, is native to southern and eastern Europe and cultivated also in countries of America and Asia [1–3]. It has been used in herbal remedies for thousands of years which dates back to ancient Egypt, Greece, and Rome [4, 5]. A diverse range of pharmacological properties have been demonstrated for this plant including anti-inflammatory [6, 7], antimicrobial [8], anticancer [9], analgesic [10], antipruritic [11], antiulcer [12], and acaricidal [13]. These pharmacological properties suggest that the plant might be a valuable therapeutic option for the prophylaxis and treatment of various diseases, including inflammation, ulcers, sedation, hemorrhoids, cough, stomach ache, pharyngitis, and rheumatic pain [14]. Previous phytochemical investigations on* M. chamomilla* have led to the isolation of essential oil, flavonoids, terpenoids, and coumarins [15–19]. Among these chemical compositions, flavonoids are the main active constituents present in the plant, which exhibited anxiolytic and antidepressant activity [20, 21]. Several methods have been developed for the determination of quercetin, luteolin, or apigenin in the plants, foods, and biological samples [22–25]. However, to our knowledge, there has been no report on the simultaneous determination of quercetin, luteolin, and apigenin in animal plasma after oral administration of* M. chamomilla *extracts.

**Figure 1:**
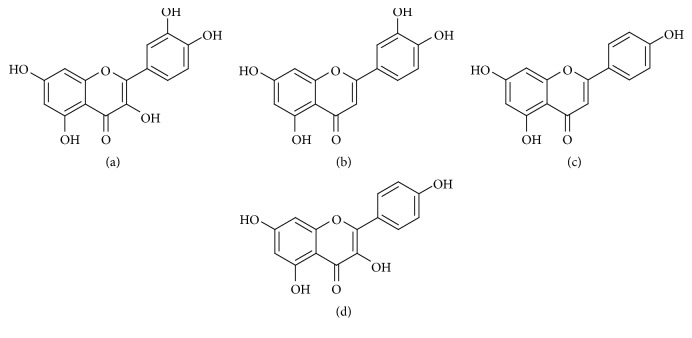
Chemical structures of quercetin (a), luteolin (b), apigenin (c), and kaempferol (d).

In this study, a simple and sensitive HPLC-UV method was first developed and validated for simultaneous determination of quercetin, luteolin, and apigenin in rat plasma. A compound, kaempferol, was selected as the internal standard (Figure 1). The method was demonstrated to be successful for application studies on pharmacokinetics of the three compounds after oral administration of* M. chamomilla *extracts. It was expected that the results of this study would provide some references to the further pharmacological study of* M. chamomilla*.

## 2. Experimental

### 2.1. Chemicals and Reagents

Reference standards of quercetin (batch number 4284, purity > 98.0%), luteolin (batch number 2226, purity > 98.0%), and apigenin (batch number 404, purity > 98.0%) were purchased from Shanghai Standard Biotech Co., Ltd. (Shanghai, China). Kaempferol (batch number 110861-200808, purity > 98.0%) was obtained from National Institutes for Food and Drug Control (Beijing, China) and used as an internal standard (IS). Methanol (HPLC-grade) was obtained from Fisher (USA). HPLC-quality water was obtained using a Cascada™ IX-water Purification System (Pall Co., USA).* M. chamomilla* was provided by the Xinjiang Medical University and authenticated by Prof. Chunsheng Liu (Beijing University of Chinese Medicine). All other reagents were of analytical grade.

### 2.2. Instrumentation and Analytical Conditions

All analyses were performed on a Shimadzu HPLC system, equipped with LC-20AT pump, a Shimadzu SCL-10A system controller, and a SPD-20A DAD-UV detector. The HPLC analysis was performed on an Agilent Eclipse XDB-C_18_ column (250 mm × 4.6 mm, 5 *μ*m) with a mobile phase consisting of methanol and 0.2% phosphoric acid (50 : 50, v/v) with a constant rate of 1.0 ml/min. The injection volume was 10 *μ*l and the column temperature was set at 25°C. The wavelength was set at 350 nm for quantitative analysis; the proportion of components in the mobile phase was optimized to obtain a well separation of quercetin, luteolin, apigenin, and kaempferol (IS).

### 2.3. Preparation of Calibration Standards and Quality Control Samples

The concentrated stock solutions of quercetin, luteolin, and apigenin were prepared by dissolving the reference standards in methanol to final concentration of 0.11 mg/ml. For the assay of plasma samples, working solutions were prepared by appropriate dilution of the stock solution with methanol. A stock solution of IS was prepared in methanol and then further diluted with methanol to prepare the working internal standard solution containing 22.52 *μ*g/ml of IS. All solutions were protected from light and stored at 4°C.

QC samples were prepared in the same way as the calibration samples, representing three different level concentrations (low, medium, and high) of quercetin in plasma at 0.57, 2.84, and 9.09 *μ*g/ml, luteolin at 0.56, 2.80, and 8.96 *μ*g/ml, and apigenin at 0.53, 2.65, and 8.48 *μ*g/ml, respectively.

### 2.4. Sample Preparation

An aliquot of 100 *μ*L rat plasma was transferred to a 1.5 mL plastic tube to which was added 10 *μ*L IS (22.52 *μ*g/ml) for processing. After being vortex-mixed for 30 seconds, the plasma sample was then extracted with 800 *μ*l of ethyl acetate and centrifuged at 18000*g* for 10 min, where ethyl acetate acted as a deproteinization-extraction agent. The procedure of extraction was performed two times. The organic layer was dried at 40°C under nitrogen stream. The residue was reconstituted in 100 *μ*L of methanol solution, vortexed for 1 min, and centrifuged at 18000*g* for 10 min. The supernatant transferred to a glass insert, and 10 *μ*L of the sample was injected into the HPLC column for analysis. The method of sample preparation was also applied for determination of accuracy, precision, and recovery.

### 2.5. Method Validation

The method was developed and conducted according to FDA guidelines with respect to specificity, linearity, LLOQ, accuracy, extract recovery, and stability.

#### 2.5.1. Specificity

The specificity study was to investigate whether endogenous constituents and other substances existing in samples will interfere with the detection of the analytes and IS. The specificity of this method was ascertained by comparatively analyzing blank plasma samples from six different sources of rats, corresponding to blank plasma spiked with the three analytes and IS and the plasma samples from the rats after oral administration of the* M. chamomilla* extract.

#### 2.5.2. Linearity and Lower Limit of Quantification (LLOQ)

The linearity of each calibration curve was determined by plotting the peak area ratio (*Y*) of analytes to the IS versus the spiked concentrations (*X*) of analytes at least six-point calibration curves. The acceptance criterion for a calibration curve was a correlation coefficient (*r*) of 0.99 or better. The LLOQ, which was defined as the lowest concentration in the calibration curve with acceptable the relative standard deviation (RSD), was within ±20% and accuracy within 100 ± 20%.

#### 2.5.3. Precision and Accuracy

The intraday precision and accuracy were assessed during the same day by analyzing six QC replicates at three levels on the same day. The interday precision and accuracy were determined by repeating analysis of QC samples on three consecutive days. The intraday and interday precisions were defined as RSD with criteria of less than 15%. The accuracy was assessed by comparing the observed concentration with its nominal value with a criterion of within ±15% for all QC samples.

#### 2.5.4. Recovery

The extraction recoveries of quercetin, luteolin, apigenin, and IS were determined by comparing the peak areas from blank plasma samples spiked with QC working solutions and IS before extraction with those from blank plasma samples spiked after extraction.

#### 2.5.5. Stability

The stability of the analytes in plasma was investigated by determining QC plasma samples of the three concentration levels under different storage conditions: 24 h at room temperature, three freeze (−20°C) and thaw (room temperature) cycles, stored at −20°C for 30 days. They were considered stable when 85–115% of the initial concentrations were got. All stability testing QC samples were determined by using the calibration curve of freshly prepared standard samples.

### 2.6. Pharmacokinetic Study

#### 2.6.1. Animals

Male Sprague-Dawley rats (body weight 160 ± 20 g) were purchased from Beijing Vital River Laboratory Animal Technology (Beijing, China). Rats were housed in a temperature- and humidity-controlled environment (25°C, 65% RH) and maintained on a 12-hour light/dark cycle for 3 days with free access to food and water before starting the experiment. The animal experiments were performed in accordance with the principles of the International Guide for the Care and Use of Laboratory Animals and were approved by the Committee on Animal Care and Usage of the Beijing University of Chinese Medicine (number SYXK 2011-0024).

#### 2.6.2. Dosing and Sampling

The extracts of* M. chamomilla* (quercetin was 8.51 mg/kg, luteolin was 56.49 mg/kg, and apigenin was 13.82 mg/kg) freshly prepared in 0.5% CMC-Na solution were orally administered to six rats. Blood samples (0.5 mL) were collected from the ocular fundus veins of rats before administration and after 10, 20, 30, and 45 min and 1, 1.5, 2, 4, 6, 8, and 12 h after oral administration of* M. chamomilla* extract. The plasma was immediately centrifuged at 3200*g* for 10 min to separate out plasma and then stored frozen at −20°C until analysis.

### 2.7. Data Analysis

The pharmacokinetic parameters of quercetin, luteolin, and apigenin were calculated by Kinetica 4.4 software (Thermo Scientific, USA). Noncompartmental analysis was used to determine standard pharmacokinetic parameters of analytes. All the results were expressed as means ± standard deviation (SD) of six replicates.

## 3. Results and Discussion

### 3.1. Method Development

#### 3.1.1. Optimization of IS

An appropriate IS will control variability in extraction and HPLC injection. In this study, several substances, such as chloromycetin, naringenin, genkwanin, and kaempferol were tested as internal standards. Among these, kaempferol has been chosen to be the most appropriate in the present analysis because it is stable and does not exist endogenously in plasma. Moreover, its retention time was suitable and it was well separated from quercetin, luteolin, and apigenin. Therefore, kaempferol was finally selected as the IS due to the relatively equal recovery, and similar polarity and retention time to the analytes.

#### 3.1.2. Optimization of Chromatographic Conditions

An Agilent Eclipse XDB-C_18_ column (250 mm × 4.6 mm, 5 *μ*m) was investigated to give an adequate separation of quercetin, luteolin, apigenin, and IS. Different compositions of mobile phase systems (acetonitrile-water, methanol-water, methanol-0.1% formic acid, and methanol-0.2% phosphoric acid) were examined and compared in order to obtain good chromatographic behavior. Finally, methanol and 0.2% phosphoric acid (50 : 50, v/v) were chosen, which had a satisfactory separation.

#### 3.1.3. Optimization of Sample Preparation Conditions

In our experiment, liquid-liquid extraction (LLE) and protein precipitation were compared for the sample preparation. Initially, we used the protein precipitation method; 1 mL methanol (or acetonitrile) was added for protein precipitation. However, the results showed that the aimed compounds could not be isolated completely from the proteins and interfering peaks in rat plasma by protein precipitation, but could be isolated by LLE. Therefore, liquid-liquid extraction by ethyl acetate was chosen, which considerably reduced the sample processing time. This method is rapid, and extraction using ethyl acetate is simple without any loss of analytes.

### 3.2. Method Validation

The developed method was validated according to FDA guidelines for the following parameters: specificity, linearity, precisions, accuracy, recovery, and stability.

#### 3.2.1. Specificity

**Figure 2:**
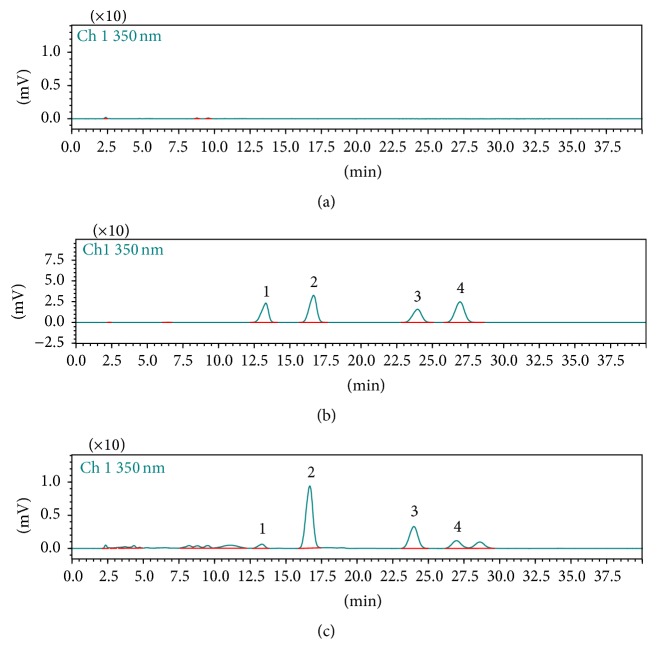
Chromatograms of quercetin (1), luteolin (2), IS (3), and apigenin (4) in plasma: (a) blank plasma; (b) blank plasma spiked with the three analytes and IS; (c) plasma sample obtained at 20 min from a rat after oral administration of* M. chamomilla *extract.

This study confirms the specificity of this method. The specificity of the method towards endogenous plasma matrix was evaluated with plasma from six rats. Typical chromatograms obtained from a blank, a spiked plasma sample with the three analytes and IS, and 20 min plasma sample after an oral administration of* M. chamomilla* extract are shown in Figure 2. As a result, the studies showed that no significant interference from endogenous substances of blank plasma was observed at the retention time of the analytes and IS.

#### 3.2.2. Linearity and LLOQ

The excellent linear relationships are shown in Table 1, where *Y* is the peak-area ratio of analytes to IS and *X* is the plasma concentration of analytes, respectively. Three calibration curves for quercetin, luteolin, and apigenin were established at six concentrations over the range of 0.11–11.36, 0.11–11.20, and 0.11–10.60 *μ*g/ml, respectively. The LLOQ for three compounds was 0.11 *μ*g/ml, which are sensitive enough for the pharmacokinetic studies of these compounds in rats.

#### 3.2.3. Precision and Accuracy

The intra- and interday precision and accuracy are demonstrated in Table 2 and investigated by analyzing QC samples. The intraday accuracy of quercetin, luteolin, and apigenin was 90.53–102.38%, 100.21–107.74%, and 94.59–104.57% with the RSD values less than 7.31%, 7.13%, and 8.32%, while the interday accuracy was 95.31–102.39%, 99.61–109.02%, and 93.17–102.36% with the RSD values less than 8.81%, 2.89%, and 2.64%, respectively. These results indicated that the developed method was precise and accurate.

#### 3.2.4. Recovery

The mean extraction recoveries and RSD for quercetin, luteolin, and apigenin from rat plasma were determined at low, medium, and high concentrations in Table 3. The recoveries of three compounds were 98.51–100.31%, 93.93–96.78%, and 92.58–97.19% with the RSD less than 9.07%, 9.97%, and 10.78%, respectively. The mean extraction recoveries for quercetin, luteolin, and apigenin were 99.11, 95.62, and 95.21%, respectively.

#### 3.2.5. Stability

Stability study showed that the concentrations of quercetin, luteolin, and apigenin were stable in plasma stored at 20°C for 24 h, −20°C for 30 days, and after three freeze-thaw cycles at low, medium, and high concentrations, respectively. All the data are summarized in Table 4.

**Figure 3:**
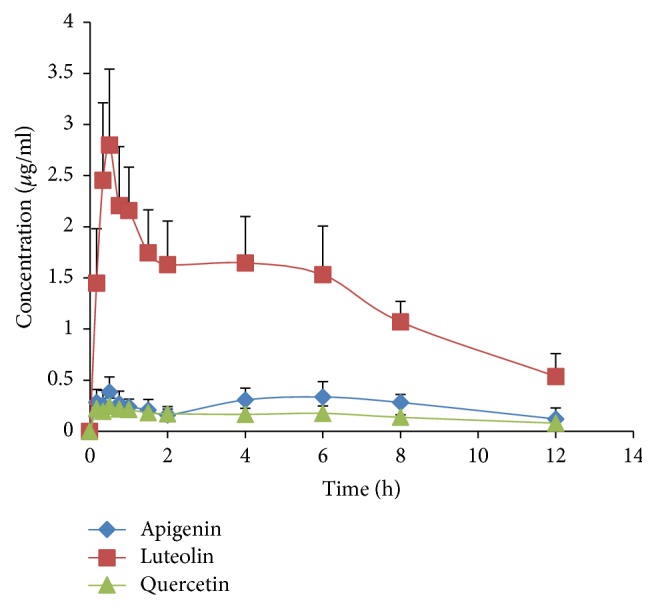
The mean (±SD) plasma concentration-time profiles of quercetin, luteolin, and apigenin after oral administration of* M. chamomilla *extract.

*Pharmacokinetics Study*. In this experiment, the method described above was applied to measure the plasma concentrations of quercetin, luteolin, and apigenin in rats administered orally with* M. chamomilla* extract. The mean plasma concentration-time curves (*n* = 6) of the analytes are shown in Figure 3. The pharmacokinetic parameters including halftime (*t*_1/2_), maximum plasma concentration (*C*_max_), time to reach the maximum concentrations (*T*_max_), and area under concentration-time curve (AUC_0–*t*_ and AUC_0–*∞*_) calculated by the noncompartment model are presented in Table 5.

The plasma concentration of quercetin reached a maximum at 0.79 h after administration with an average *C*_max_ of 0.29 *μ*g/ml. The area under the curve (AUC_0–*∞*_) was 3.88 *μ*g h/ml. The plasma concentration of luteolin reached a maximum at 0.42 h after administration with an average *C*_max_ of 3.04 *μ*g/ml. The area under the curve (AUC_0–*∞*_) was 19.89 *μ*g h/ml. The plasma concentration of apigenin reached a maximum at 0.51 h after administration with an average *C*_max_ of 0.42 *μ*g/ml. The area under the curve (AUC_0–*∞*_) was 5.03 *μ*g h/ml.

## 4. Discussion

Quercetin, luteolin, and apigenin are the main active constituents present in the* M. chamomilla* extract, which exhibited anxiolytic and antidepressant activity. However, little data were available regarding the pharmacokinetic characterization of quercetin, luteolin, and apigenin in rats after oral administration of* M. chamomilla* extract. This is the first report of HPLC-UV quantitative assay for investigating the pharmacokinetics of* M. chamomilla* extract. There were no interferences from endogenous substances. Pharmacokinetic results showed that the three compounds reached a maximum at about 0.79 h, 0.42 h, and 0.51 h, respectively, indicating that they may be absorbed quickly after oral administration. However, the elimination half-life (*T*_1/2_) was about 13.60 h, 4.43 h, and 8.82 h, respectively, suggesting that the elimination of them may be slow in rats after oral administration f* M. chamomilla* extract.

## 5. Conclusion

The present study described a simple, specific, and reliable HPLC-UV with IS method for the simultaneous determination of three flavonoids in blood samples. This is the first validated HPLC method for analysis of quercetin, luteolin, and apigenin in rat plasma, which was highly sensitive and accurate. The method has been successfully applied to pharmacokinetic studies of quercetin, luteolin, and apigenin in rats after oral administration of* M. chamomilla *extract. The pharmacokinetic results would be a suitable reference in clinical application of* M. chamomilla*.

## Figures and Tables

**Table 1:** Linearity and LLOQ for the analysis of quercetin, luteolin, and apigenin under standard solutions.

Compound	Linearity (*μ*g/ml)	Calibration curve	Correlation coefficient (*r*)	LLOQ (*μ*g/ml)
Quercetin	0.11–11.36	*Y* = 0.37*X* − 0.03	0.99	0.11
Luteolin	0.11–11.20	*Y* = 0.62*X* − 0.01	0.99	0.11
Apigenin	0.11–10.60	*Y* = 0.63*X* + 0.05	0.99	0.11

**Table 2:** The intra- and interday precision and accuracy of quercetin, luteolin, and apigenin in rat plasma (*n* = 6).

Compounds	Spiked conc. (*μ*g/ml)	Intraday			Interday		
		Found conc. (*μ*g/ml)	Precision (RSD%)	Accuracy (%)	Found conc. (*μ*g/ml)	Precision (RSD%)	Accuracy (%)
Quercetin	0.57	0.58 ± 0.04	7.31	102.38	0.58 ± 0.01	2.20	102.39
	2.84	2.57 ± 0.11	4.22	90.53	2.81 ± 0.25	8.81	99.05
	9.09	8.79 ± 0.61	6.99	96.67	8.66 ± 0.11	1.31	95.31
Luteolin	0.56	0.60 ± 0.02	2.79	107.74	0.61 ± 0.01	1.06	109.02
	2.80	2.81 ± 0.20	7.13	100.32	2.90 ± 0.08	2.89	103.68
	8.96	8.98 ± 0.53	5.90	100.21	8.92 ± 0.05	0.54	99.61
Apigenin	0.53	0.50 ± 0.04	8.32	94.59	0.49 ± 0.01	2.64	93.17
	2.65	2.77 ± 0.15	5.50	104.57	2.71 ± 0.05	1.88	102.36
	8.48	8.46 ± 0.52	6.12	99.72	8.41 ± 0.04	0.47	99.23

**Table 3:** Extraction recovery of quercetin, luteolin, and apigenin in rat plasma (*n* = 6).

Compounds	Spiked conc. (*μ*g/ml)	Recovery (%)	RSD (%)	Average (%)
Quercetin	0.57	98.52 ± 6.97	7.08	99.11
	2.84	100.31 ± 9.04	9.01	
	9.09	98.51 ± 8.93	9.07	
Luteolin	0.56	96.15 ± 9.22	9.58	95.62
	2.80	96.78 ± 4.15	4.29	
	8.96	93.93 ± 9.36	9.97	
Apigenin	0.53	95.87 ± 10.13	10.56	95.21
	2.65	97.19 ± 4.34	4.47	
	8.48	92.58 ± 9.98	10.78	

**Table 4:** Stability of quercetin, luteolin, and apigenin in rat plasma (*n* = 6).

Compounds	Spiked conc. (*μ*g/ml)	Short-term stability (24 h at room temperature)		Long-term stability (30 days at −20°C)		Freeze-thaw stability (3 cycles)	
		Found conc. (*μ*g/ml)	RSD (%)	Found conc. (*μ*g/ml)	RSD (%)	Found conc. (*μ*g/ml)	RSD (%)
Quercetin	0.57	0.63 ± 0.01	1.30	0.62 ± 0.03	5.16	0.63 ± 0.01	2.08
	2.84	3.09 ± 0.18	5.68	3.10 ± 0.16	5.17	3.09 ± 0.09	2.78
	9.09	9.15 ± 0.69	7.52	9.59 ± 0.53	5.52	9.24 ± 0.55	5.97
Luteolin	0.56	0.62 ± 0.01	1.21	0.61 ± 0.03	5.52	0.62 ± 0.02	3.01
	2.80	2.94 ± 0.14	4.65	2.92 ± 0.15	5.03	2.91 ± 0.15	5.23
	8.96	8.96 ± 0.76	8.53	8.99 ± 0.65	7.28	8.56 ± 0.54	6.33
Apigenin	0.53	0.50 ± 0.04	8.36	0.46 ± 0.04	8.47	0.48 ± 0.02	3.21
	2.65	2.64 ± 0.16	6.14	2.61 ± 0.13	5.07	2.60 ± 0.14	5.31
	8.48	8.31 ± 0.72	8.61	8.27 ± 0.62	7.46	7.87 ± 0.50	6.39

**Table 5:** Pharmacokinetic parameters of quercetin, luteolin, and apigenin in rats after oral administration of *M. chamomilla *extract (mean ± SD, *n* = 6).

Parameters	Unit	Quercetin	Luteolin	Apigenin
*T*_max_	h	0.79 ± 0.25	0.42 ± 0.09	0.51 ± 0.13
*C*_max_	*μ*g/ml	0.29 ± 0.06	3.04 ± 0.60	0.42 ± 0.11
AUC_0–12_	*μ*g h/ml	1.79 ± 0.29	16.00 ± 2.38	2.91 ± 0.74
AUC_0–*∞*_	*μ*g h/ml	3.88 ± 1.00	19.89 ± 5.06	5.03 ± 1.72
*T*_1/2_	h	13.60 ± 6.62	4.43 ± 1.84	8.82 ± 4.15

Parameters were estimated using the mean concentration-time profiles obtained from six different rats per time point (*n* = 6).
